# Nature versus art as elicitors of the sublime: A virtual reality study

**DOI:** 10.1371/journal.pone.0233628

**Published:** 2021-03-22

**Authors:** Alice Chirico, Robert R. Clewis, David B. Yaden, Andrea Gaggioli

**Affiliations:** 1 Department of Psychology, Università Cattolica del Sacro Cuore di Milano, Milan, Italy; 2 Department of Philosophy, Gwynedd Mercy University, Gwynedd Valley, PA, United States of America; 3 Max Planck Institute for Empirical Aesthetics, Frankfurt am Main, Germany; 4 Department of Psychology, University of Pennsylvania, Philadelphia, PA, United States of America; 5 Department of Psychiatry and Behavioral Sciences, Johns Hopkins Medicine, Baltimore, Maryland, United States of America; 6 ATNP-Lab, Istituto Auxologico Italiano, Milan, Italy; National University of Singapore, SINGAPORE

## Abstract

The sublime–the mixed aesthetic experience of uplift and elevation in response to a powerful or vast object that otherwise is experienced as menacing–has nurtured philosophical discourse for centuries. One of the major philosophical issues concerns whether the sublime is best thought of as a subjective response or as a stimulus. Recently, psychology has conceived of the sublime as an emotion, often referred to as awe, arising from natural or artistic stimuli that are great, rare, and/or vast. However, it has not yet been empirically demonstrated whether two major elicitors of the sublime–nature and art–differ in inducing this state. In order to experimentally compare nature and art, we exposed 50 participants to sublimity-inducing content in two different formats (nature-based and art-based) using 360° videos. We compared Vincent Van Gogh’s *The Starry Night* with a photorealistic version of the actual place depicted in the painting, Saint-Rémy-de-Provence. We measured participants’ emotional responses before and after each exposure, as well as the sense of presence. The nature-based format induced higher intensity emotional responses than the art-based format. This study compares different sublime stimuli (nature vs. art) for eliciting the sublime.

## Introduction

Imagine first the most awe-inspiring natural scenery that you have ever seen, generally involving a grand and sweeping panorama. Then, imagine viewing a masterful painting of the same scene. One can imagine similarities and differences between one’s reactions to these two scenes. Both the real natural scenery and the painting of it would likely display similar physical properties, such as apparent vastness, rarity, and novelty. These features are crucial for the emergence of a particular mental process traditionally called the sublime or (equivalently) sublimity [e.g., 1–3] or, in more recent years, awe [4–10]. However, these scenes would differ, too, because one would know that one elicitor is real and the other is a representation. Does this distinction between ‘real’ and ‘representation’ matter when it comes to experiencing the sublime?

This question touches on a long-standing debate on the nature of sublime as an object (i.e., elicitor) or as a mental process (i.e., a subjective state experienced while reacting to a given elicitor) (see [11] for a brief review). While the philosophical debate between characterizing the sublime as stimuli or mental state is on-going, psychologists have tended to treat the sublime as a mental state or, more specifically, as an emotion [12–14]. An increasing number of psychologists have started to think of the emotion of awe as the psychological counterpart to the sublime in philosophical aesthetics [e.g., 5, 15, 16]. The sublime has been characterized (following Burke) as a state of amazement tinged with fear or at least some negative components [13]. The elicitors are typically characterized as grand, rare, novel, and vast [13]. On the other hand, the nature of objects at the basis of the sublime requires further elucidation [12], in order to define a “limited theory” [16] (p.3) which allows for a more precise operationalization of the sublime [16, 17].

There is a deep-rooted philosophical debate regarding the relative merits of different elicitors of the sublime [e.g., 3, 18–21]. Several scholars, including Kant and Burke, prioritized nature over art [e.g., 19, 22, 23] or at least painting (Burke thought poetry was an effective stimulus). Burke and Kant provided several guidelines to “design” sublime stimuli. Burke accepted that sublimity and beauty could be combined in the same object, even if they are distinct. Burke also listed many properties natural stimuli should possess to elicit the sublime such as being vast or fearsome (terrifying). Kant [3] built upon and developed this work by considering two kinds of sublimity. One kind is elicited by natural stimuli perceived to be much more powerful than us (i.e., the dynamical sublime), which is similar to the Burkean sublime. Another, more original kind introduced by Kant, is elicited by something that is so vast that it seems unable to be taken in or grasped by our senses or imagination (i.e., the mathematical sublime). Conversely, Hegel [24] and Longinus himself [25] (author of the first extant treatise on the sublime) considered poetry and rhetorical speech to be the main elicitors of the sublime.

Contemporary experimental researchers have tended to use representations of the sublime of natural settings, such as landscapes or storms, to elicit the sublime in the lab [11, 13, e.g.]. Only a few researchers have focused on art-based elicitors of the sublime [e.g., 1]. Researchers have typically relied on pictures or videos to evoke the sublime. So far, no studies have tested whether differences in subjective experience arise from different types of elicitors.

In this study, we examined differences between a nature-based or an art-based sublime-eliciting stimulus by means of Virtual Reality 360° videos, which provides a high level of ecological validity even in a constrained space. In this regard, we have recently demonstrated that participants exposed to the same naturalistic content displayed through 360° videos and in reality, showed the same emotional profile related to the experience. Chirico and Gaggioli [26] showed that a 360° video of a natural scenario (i.e., Iseo Lake in Italy) was equivalent to the corresponding scenario (of the real Iseo Lake), at least, at the emotional level. This motivated our comparison between a nature-based sublime and an artistic-based sublime in this study using 360° videos.

We took advantage of a unique property of 360° videos technology. This technology allows content to be displayed either in an artistic or in a naturalistic format. In terms of the specific content, we chose *The Starry Night* by Van Gogh (1889). We chose this particular image for two main reasons. First, this painting has been determined to fit the criteria of being sublime by a number of scholars [e.g., 27, 28]. Second, this painting is based on a real scene, the village of Saint-Rémy-de-Provence before sunrise, so we could show a nature-based version of the actual scene depicted in this painting. We compared the effects of a nature-based and an art-based format in terms of their relative impact on the subjective state of the sublime. Drawing on philosophical and psychological work on the sublime [2, 16, 29], we may expect nature-based elicitors to appear more threatening or to hold, at least, a blended or mixed emotional profile, as suggested mainly by Burke’s account [2]. Although an early study of the link between sublime and fear in artworks showed that fear predicted a feeling of the sublime [30], this has since been questioned by Hur and colleagues [16], who conclude that whether fear is a component of the sublime merits further study. Furthermore, both sublime art and sublime nature are thought to be perceived as “transcendent” and “perceived or imagined in a new light, in a rare moment” [31].

Moreover, in line with Keltner and Haidt’s model on awe [5] and recent models on awe [9, 10, 32], which differentiate perceptual and conceptual vastness, one might have thought that the sublime in natural landscape would be perceived as more perceptually vast, while art-based sublimity would be experienced more as conceptually vast, i.e., cognitively complex. However, no empirical evidence exists with this regard. Both formats can be expected to trigger a disposition to share the experience with others [31], thus, no difference between the two formats should emerge with regard to this aspect. Past theories have tended to describe it as a personal, even lonesome experience [e.g., 3], but current theories defend the experience’s normative character and intersubjectivity and emphasize that the sublime may be shared and communicated with others [12]. Finally, following Burke and Kant, beauty is conceived as being conceptually distinct from and a counterpart to the sublime.

To conclude, despite this traditional differentiation between natural and artistic elicitors, the above-mentioned key dimensions should be considered as part of both forms of the sublime. We describe in detail these central dimensions as follows: (i) rarity (see 1, 34); (ii) beauty perception (as a reverse: see [2]); (iii) self-transcendence [8]; (iv) conceptual complexity [5]; (v) perception of existential danger (Burke) [2]; (vi) perception of vastness [5], which is related to the psychological counterpart of the sublime, that is, awe; (v) need to share (the experience) [12]; (vii) existential safety [1, 33]. Since these features have been considered as cross-dimensions of different sublime types, we intended to keep the study exploratory regarding these dimensions of the sublime. Therefore, a definitive null/alternative hypothesis regarding the intensity of sublime and sub-dimensions of the sublime can be formulated as follows:

H_0_: Art-based and Nature-based formats are equal in terms of the intensity of the sublime and sublime subdimensions.
H_1_: Art-based and Nature-based formats differ in terms of the intensity of the sublime and sublime subdimensions.

In this study, we chose a Bayesian approach to test the null hypothesis against the alternative to obtain a ratio of the probability of the strength of each hypothesis.

The aim of this work is to address if, how, and to what extent an art-based format and a nature-based format could differ in eliciting the sublime.

## Methods

### Sample

We involved 50 participants (39 females), mean age = 24.70 (SD = 4.703), from Lombardy, a region of Italy, who voluntarily took part in the research. The average number of years in education was 17.04 (S.D. = 2.23). Participants who (at the time of the experiment) reported vestibular and/or balance disorders were excluded. The experimental protocol was approved by the Ethical Committee of the Università Cattolica del Sacro Cuore prior to data collection. Each participant provided written informed consent for study participation. The whole procedure was carried out in accordance with the Helsinki Declaration.

### Materials and procedure

Participants underwent a within-subject design in which they were randomly assigned to both of the following conditions in a counterbalanced order:

- Nature-based condition: participants watched a 360° video panorama view of *Saint-Rémy-de-Provence*, that is, the subject of *The Starry Night* expressionist [34] painting by Van Gogh, created by means of Ricoh Theta S, and using ShotCut video-editing free online tool.
- Art-based condition: participants watched a 360° video of the painting *The Starry Night* by Van Gogh [35].

After providing consent and completing demographic questions, participants were asked to sit on a chair and to report the extent to which they experienced nine discrete emotions (i.e., anger, disgust, fear, pride, amusement, sadness, joy, beauty, sublime) on a 10-point Likert scale which has been used in previous research on aesthetic emotions in real and virtual spaces [26, 34, 36–38] and the Positive and Negative Affective Schedule [39] to control for the pre-experimental affective states. Crucially, items related to beauty and sublime have been added *ad hoc* for the purpose of this study. Participants then put on a VR Head Mounted Display (Gear VR) combined with a smartphone (i.e., Galaxy Note 4). Participants were provided with standardized instructions about how to make the video start (4:26 minutes long) using this virtual reality device (for more details on this procedure, see [38, 40]). After the exposure to the nature-based or art-based video, participants rated again the extent to which they experienced nine discrete emotions combining both basic and aesthetic emotions (i.e., anger, disgust, fear, pride, amusement, sadness, joy, beauty, sublimity) [26, 34, 36–38], positive and negative affect [39] and the sense of presence (i.e., the feeling of being ‘there’ in the virtual environment [41], through the ITC-Sense of Presence Inventory (ITC-SOPI) [42]. ITC-SOPI is a 36-item questionnaire assessing four dimensions of the sense of presence (i.e., sense of physical presence; engagement; ecological validity, negative effects) on a 5-point Likert scale (1 = strongly disagree to 5 = strongly disagree). Moreover, participants’ disposition to live positive emotions (Disposition Positive Emotions Scale)–DPES–[43] and their general aesthetic interest for literature, art, cinema, design, food and nature (Desire for Aesthetics Scale-DFAS) [44] was assessed across 36 item on a 6-point Likert scale. A score of 216 indicates a strong interest in aesthetics, while a score of 144 reflects mild and a score of 108 a neutral aesthetic interest. Finally, to disambiguate the effect of the two elicitors on the experiential profile of the sublime, we developed 40 *ad hoc* sublime items drawing from Burke, Kant, Konečni, and Clewis on the sublime as well as the Keltner and Haidt model of awe (i.e., in which the sublime was conceived as an “awe-like aesthetic emotion”) on a 7-point Likert scale (1 = not at all; 7 = at all). Each item tapped into a specific dimension of the sublime presented in the Introduction section: (i) rarity [1, 33, see]; (ii) beauty perception (as a reverse: see [2]); (iii) self-transcendence [8]; (iv) conceptual complexity [5]; (v) perception of existential danger (Burke) [2]; (vi) perception of vastness [5], which is related to the psychological counterpart of the sublime, that is, awe; (v) need to share (the experience) [12]; (vii) existential safety [1, 33].

The questionnaire has not been previously validated, but all information on participants is reported within this study in the results section as well as in the S1 File.

## Results

### Data analysis

First we calculated descriptive statistics for each discrete emotion, affect and sense of presence dimensions in both conditions. Then, since not all of our emotion-related variables were normally distributed and since we aimed to test both differences and similarities among stimuli, we opted for a Bayesian approach of analyses across all measures. Bayes factors provide and rely on the likelihood of both the null and the alternative hypothesis. Thus, our analyses not only allowed us to test the null hypothesis, but we could also estimate the likelihood of both null and alternative hypotheses. Specifically, we carried out a repeated measure Bayesian t-test to compare both conditions (i.e., nature-based and art-based) regarding each emotion (i.e., anger, disgust, fear, pride, amusement, sadness, joy, beauty, sublimity), general affect (PANAS) [see Table 1], dimensions of the sense of presence (i.e., sense of physical presence; engagement; ecological validity, negative effects), and each dimension of the *ad hoc* sublime scale. Finally, we conducted an ANCOVA with sublime as a measure as well as the disposition to experience each positive emotions (DPES sub-dimensions) and the disposition to seek aesthetic experiences (DFAS dimensions) as covariates.

**Table 1:** Bayesian t-test dependent samples analyses.

	Bayesian paired sample t-test within conditions		
Variable	BF_10_	err	Effect supposed for H_1_
**Anger**	2.641	383e -6	Anecdotal^a^
**Disgust**	0.246	2.881e -6	Insignificant*
**Fear**	433.304	3.711e -9	**Decisive**^a^
**Pride**	0.226	2.771e -6	Insignificant
**Amusement**	0.163	2.121e -6	Insignificant
**Sadness**	0.225	2.765e -6	Insignificant
**Joy**	0.448	3.093e -6	Insignificant
**Beauty**	0.194	2.506e -11	Insignificant
**Sublime**	0.164	2.126e -6	Insignificant
**Positive Affect**	82.281	4.242e -8	**Decisive**
**Negative Affect**	0.158	4.533e -6	Insignificant
**Physical presence**	1621	2.59e-07	**Decisive**
**Ecological Validity**	100.128.094	1.70e-05	**Decisive**
**Engagement**	1.499	1.99e-03	Anecdotal
**Negative Effect**	12.62	3.62e-04	**Strong**^a^

^*a*^ Effect terminology from Jeffreys and Jeffreys [45].

* = Authors’ term to indicate a negligible probability of H_1_ with respect to H_0_; it does not refer to the significance of differences between means.

### Descriptive statistics

**Fig 1:**
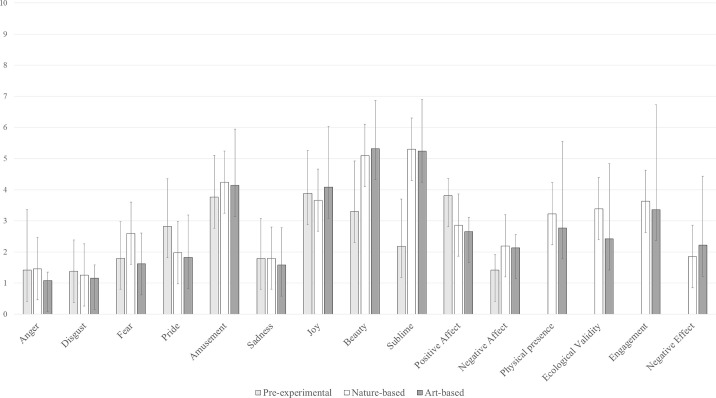
Descriptive statistics for emotions, affect, and sense of presence dimensions. Mean participants’ ratings of each discrete emotion and affect before and after each experimental condition. Mean participants’ ratings of sense of presence dimensions after each experimental condition. Error bars represent standard deviations.

Mean and standard deviation for all discrete emotions, affect and sense of presence dimensions were computed (Fig 1) and numerical data are reported in the S1 File.

### Bayesian t-test dependent samples analysis on discrete emotions, affect and sense of presence dimensions

An estimated Bayes factor (null/alternative) suggested that anger, fear, general positive affect, sense of physical presence, ecological validity, were highly in favor of the alternative hypothesis according to the direction indicated by the mean values. All other likelihood values did not indicate a high probability that the two stimuli differed regarding each of the remaning emotional dimensions, Table 1

### Sublime dimensions

We computed both Bayesian t-test for null hypothesis (BF_01_) and classical paired sample t-test for each dimension of the sublime scale that we developed *ad hoc* for this study. Specifically, first, we compared the art-based format with the nature-based format regarding each of the sublime dimensions (i.e., rarity; beauty perception; self-transcendence; conceptual complexity; perception of existential danger; perception of vastness; need to share the experience; existential safety) following a Bayesian approach of analysis. The dimension that showed evidence for a difference (in favor of the alternative hypothesis) between the art and nature-based content was the perception of *vastness* (BF_01_ = 1.033; err. = 2.408e-6) along with the dimension of *perception of existential danger* (BF_01_ = 3.961e -5; S.D. = 4.162e -7). Specifically, nature-based content elicited a higher sense of *vastness* (mean = 29.40; S.D. = 7.279) and *perception of existential danger* (mean = 22.34; S.D. = 7.69) compared to the art-based one (*vastness*: mean = 27.18; S.D. = 8.186) (*perception of existential danger*: mean = 16.38; S.D. = 5.721). Both *vastness* and p*erception of existential danger* showed no evidence for H_0_ (i.e., strong evidence for H_1_). Then, we also conducted paired sample t-test for each sublime dimension, comparing the art-based format with the nature-based format, Table 2.

**Table 2:** Paired sample t-test and descriptive statistics for sublime dimensions in both conditions.

				Descriptive Statistics a and paired sample t-test						
	Bayesian t-test dependent samples			Art-based		Nature-based		Nature vs. Art		
**Sublime dimensions**	**BF**_**01**_	**err**	**Effect supposed for H**_**0**_	**Mean**	**SD**	**Mean**	**SD**	**t**	**p**	***d* Cohen**
**Rarity**	6.304	2.048e -6	Strong	25.9	5.5997	25.72	4.8741	.253	.801	0.0358
**Beauty Perception**	6.479	1.975e -6	Strong	26.18	7.1105	26.22	6.864	-.030	.976	-0.0042
**Self-Transcendence**	6.121	2.123e -6	Strong	26.32	5.6657	26.68	5.7016	-.355	.724	-0.0502
**Conceptual Complexity**	1.171	2.61e-6	Anecdotal	19.6	6.1246	21.58	6.6856	-1.932	.059	-0.2732
**Perception of Existential Danger**	3.961e -5	4.162e -7	**No evidence**	16.38	5.721	22.34	7.6894	-5.720	.**000**	-0.809
**Perception of Vastness**	0.968	2.408e -6	**No Evidence**	27.18	8.1858	29.4	7.2787	-2.041	**.047**	-0.2886
**Need to Share**	1.411	2.794e -6	Anecdotal	26.66	5.0127	28	4.8234	-1.821	.075	-0.2575
**Existential Safety**	1.140	2.585e -6	Anecdotal	22.18	5.271	20.5	5.8771	1.948	**.057**	0.2755

Descriptive statistics of sublime dimensions in the two conditions, “Art-based” and “Nature-based” sublime, Bayesian t-test dependent samples analyses for each sublime dimension and classical paired samples t-test comparisons for each sublime dimension in both conditions. In bold, significant results.

### Dispositional variables

Finally, we centered all DPES dimensions (Joy, Contentment, Pride, Compassion, Love, Amusement, Awe) and DFAS and included each as covariate in a within-subject ANOVA with sublime as a measure and condition as the independent variable. No effect of any covariate was found. DPES scores in this Italian sample were as follows: Joy (mean = 29.18; S.D. = 5.84); Contentment (mean = 24.24; S.D. = 4.32); Pride (mean = 26.94; S.D. = 3.74); Compassion (mean = 28.26; S.D. = 3.78); Love (mean = 27.24; S.D. = 5.42); Amusement (mean = 25.92; S.D. = 5.24); Awe (mean = 27.22; S.D. = 4.40). DFAS scores ranged from a minimum of 103.00 to a maximum of 15, with a mean score of 127.82 (S.D. = 10.91).

## Discussion

Grounded in a centuries-old literature on the sublime and drawing from recent psychological research on this emotion, this study compared the two longest-lasting sublime-eliciting formats (i.e., nature and art) under controlled conditions using 360° immersive videos. The scene depicted was nearly the same, but conditions differed in terms of whether the scene was photorealistic or a painting, that is *Saint-Rémy-de-Provence* or *The Starry Night* by Van Gogh. The subjective experience of the sublime differed in several ways. First, the nature-based format created more feelings of being present in the simulated scenario compared to the art-based format. The art-based format was more likely to create negative effects related to 360° video (i.e., dizziness and disorientation).

Moreover, we found that the stimuli were similar in terms of most emotions elicited, except for fear, as a discrete emotion, and positive affect, as a general affect dimension. The nature-based format resulted in significantly higher fear and positive affect compared to the art-based format. This could be explained in relation to the nature of the Burkean sublime as a mixed emotional feeling tinged with fear or sense of danger [13, 15, 29]. Results suggested that this last element was prevalent in the nature-based format, in line with Burkean theorization of the sublime [2] and with the empirical findings of Hur and coll [13].

Importantly, the two stimuli, considered as elicitors of the sublime, reported a likelihood in favor of the alternative hypothesis that was due to chance. They did not differ in terms of their ability to elicit an experience of the sublime. However, some differences regarding specific dimensions of the sublime measured by the authors’ *ad hoc* questionnaire were found. Regarding the single dimensions of the sublime “Perception of existential danger” and “Perception of vastness” were higher in the nature-based format compared to the art-based. The nature-based format was perceived as more perceptually vast and as harbinger of possible existential danger. Conversely, the art-based format elicited a higher sense of being in a “safe” existential condition.

Moreover, the two formats did not differ in relation to the target measure of the sublime, neither after including dispositional variables concerning Positive Emotion Dispositions (DPES single dimensions) nor the Desire for Aesthetics (as measured by DFAS). This result may suggest the overarching power of the sublime to overcome intra-individual differences related to stable personal traits. The sublime might be a matter of context and stimulus more than of predisposition or tendencies, since specific emotion dispositions and personal desire for aesthetic dimensions did not impact on the sublime experience after nature and art exposure. However, whether personality traits or other stables dispositions, such as cognitive styles, could impact on people’s ability to experience the sublime needs to be investigated in future studies.

Despite the limited number of participants and the preliminary nature of the measures and the approach, this study added to the pre-existing literature into three distinct ways. First, it addressed a long-lasting philosophical question regarding the nature of sublime elicitors, by framing it within the paradigm of experimental psychology. Then, it suggested that both formats were able to elicit the sublime at the same intensity, but in different ways. The artistic format resulted in an increased feeling of existential safety when compared to the naturalistic one. Conversely, the nature-based format resulted in higher fear but not general overall negative affect compared to the art-based one, suggesting that this specific negative emotional component, fear, plays a key role in the experience of the sublime, but a role whose relation to the overall positive affect of the sublime experience is not fully understood.

## Conclusions

This study showed the potential of immersive media, such as 360° videos, to investigate complex research questions and phenomena in a controlled setting. There was no significant difference with regard to the capacities of art-based and nature-based stimuli to evoke the experience of the sublime.

Finally, despite our participants had previous knowledge of Starry Night by Van Gogh and had already seen a landscape similar to that showed in the naturalistic format, they reported high sublime scores compared to other emotions. A useful future step could be to test whether previous experiences with, and knowledge of the sublime-eliciting stimuli significantly impacts participants’ sublime reports and/or on psychophysiological reactions. We expect technologies to continue to create ever more immersive experiences, providing new opportunities to investigate the centuries old and still on-going philosophical and psychological discourses on the sublime.

## Supporting information

S1 File (DOCX)
