# Supplementary material for: Nature versus art as elicitors of the sublime: A virtual reality study
Source: PLoS One. 2021 Mar 22;16(3):e0233628. doi: 10.1371/journal.pone.0233628 (PMC7984734; doi:10.1371/journal.pone.0233628)
Supplement: S1 File — (DOCX) [file pone.0233628.s001.docx]

**Nature and Art as Elicitors of the Sublime: A Virtual Reality Study**

Alice Chirico^1*^, Robert R. Clewis^2,3^, David B. Yaden,^4^ & Andrea Gaggioli^1,5^

^1^Università Cattolica del Sacro Cuore di Milano; Department of Psychology

^2^ Gwynedd Mercy University,

^3^Max Planck Institute for Empirical Aesthetics, Philosophy

^4^ University of Pennsylvania, Department of Psychology

^5^ Istituto Auxologico Italiano, ATNP-Lab

**S1. *Ad hoc* Questionnaire on the Sublime**

***7-point Likert scale (from 1 = not at all to 7 = very much)***

***RARENESS***

***(Percezione Di Rarità):***

1. ***I perceived as if I have already lived an experience similar to this one.* (R)^[[1]](#footnote-1)^**

(Ho percepito di aver già vissuto un’esperienza di questo tipo)

1 2 3 4 5 6 7

1. **I feel that an experience like this can happen to me again (R)**

(Ritengo che un’esperienza come questa possa accadermi nuovamente).

1 2 3 4 5 6 7

1. **Even though I have already lived this kind of experience, I feel as if it is new**.

(Pur avendola già vissuta, percepisco questa esperienza come unica nel suo genere).

1 2 3 4 5 6 7

1. **I did not perceive this experience as unusual (R)**

(Non ho percepito questa esperienza come insolita)

1 2 3 4 5 6 7

1. **I experimented something extremely new**.

(Ho sperimentato qualcosa di estremamente nuovo).

1. 2 3 4 5 6 7
2. **I think this is a sporadic experience.**

(Penso che questa esperienza sia un’esperienza sporadica).

1 2 3 4 5 6 7

***BEAUTY PERCEPTION***

***(Percezione Del Senso Di Bellezza):***

- - - 1. **I experienced contents as beautiful.**

(I contenuti che ho esperito sono belli).

1 2 3 4 5 6 7

- - - 1. **I felt attracted by what I was experiencing.**

(Mi sentivo attratto/a da ciò che esperivo).

1 2 3 4 5 6 7

- - - 1. **I felt fascinated by what I was seeing.**

(Mi sono sentita incantato/a da ciò che vedevo).

1 2 3 4 5 6 7

- - - 1. **I think that the environment I saw showed a perfect balance among its dimensions.**

(Penso che l’ambiente esperito sia in perfetto equilibrio tra le sue dimensioni).

1 2 3 4 5 6 7

- - - 1. **I felt involved in the environment.**

(Mi sono sentito coinvolto/a nell’ambiente mostrato).

1 2 3 4 5 6 7

***SELF-TRANSCENDENCE***

***(Auto-Trascendenza)***

1. **I lost contact with the surrounding reality.**

(Ho perso il contatto con la realtà che mi circondava).

1 2 3 4 5 6 7

1. **I felt as if I was in a trance-like state**.

(Mi sono sentito come in uno stato di trance).

1 2 3 4 5 6 7

1. **I felt connected with all the surroundings.**

(Mi sono sentito connesso a tutto ciò che mi circondava).

1 2 3 4 5 6 7

1. **I felt I was deeply alone. (R)**

(Ho sentito di essere profondamente solo/a).

1 2 3 4 5 6 7

1. **I felt I was detached from the rest of the world**. **(R)**

(Ho sentito che ero scollegato rispetto a tutto il resto del mondo).

1 2 3 4 5 6 7

1. **I felt my time perception changing.**

(Ho sentito cambiare la percezione del tempo).

1 2 3 4 5 6 7

***CONCEPTUAL COMPLEXITY***

***(Complessità Concettuale)***

1. **I struggled to find a rational explanation for what was going on.**

(Faccio fatica a dare una spiegazione razionale a ciò che ho sperimentato).

1 2 3 4 5 6 7

1. **I deeply understood what I experienced.** **(R)**

(Ho compreso fino in fondo quello che ho esperito).

1 2 3 4 5 6 7

1. **I feel I am able to describe what I lived.** **(R)**

(Penso di essere in grado di descrivere ciò che ho vissuto).

1 2 3 4 5 6 7

1. **I feel I did not understand the final aim of the experience I lived.**

(Penso di non aver capito il fine ultimo dell’esperienza che ho vissuto).

1 2 3 4 5 6 7

1. **I think I am able to find an explanation for what I lived. (R)**

(Penso di essere in grado di giustificare ciò che ho vissuto).

1 2 3 4 5 6 7

1. **I struggled to understand the complexity of what I experienced.**

(Mi sono sentito in difficoltà nel cercare di comprendere la complessità di ciò che ho sperimentato).

1 2 3 4 5 6 7

***PERCEPTION OF EXISTENTIAL DANGER***

***(Percezione di Pericolo Esistenziale)***

1. **I was afraid of the environment I saw.**

(Mi sono sentito in soggezione dall’ambiente che ho esperito).

1 2 3 4 5 6 7

1. **I felt impotent in front of the environment I saw.**

(Mi sono sento impotente rispetto all’ambiente che ho esperito).

1 2 3 4 5 6 7

1. **I felt defenceless.**

(Mi sono sentito inerme).

1 2 3 4 5 6 7

1. **I felt overwhelmed by something bigger than me.**

(Mi sono sentito sovrastato da qualcosa di più grande di me).

1 2 3 4 5 6 7

1. **I felt able to respond to the environment I saw. (R)**

(Mi sono sentito all’altezza di reagire dinnanzi all’ambiente che ho esperito).

1 2 3 4 5 6 7

1. **I wished to interrupt the experience.**

(Avrei voluto interrompere la mia esperienza).

1 2 3 4 5 6 7

***PERCEPTION OF VASTNESS***

***(Percezione di Vastità)***

1. **I felt as if I was part of something bigger than me.**

(Ho percepito di essere inserito in qualcosa molto più grande di me).

1 2 3 4 5 6 7

1. **I understood the real meaning of vastness.**

(Ho conosciuto il vero significato della grandiosità).

1 2 3 4 5 6 7

1. **I perceived the stimulus in front of me as vast compared to me.**

(Ho percepito lo stimolo che avevo davanti come vasto rispetto a me).

1 2 3 4 5 6 7

1. **I felt immersed in an environment much bigger than me.**

(Mi sono sentito inserito in un ambiente molto più grandioso di me).

1 2 3 4 5 6 7

1. **I did not feel the vastness of the experience I lived.**

(Non ho percepito della grandezza nell’esperienza che ho vissuto).

1 2 3 4 5 6 7

1. **I felt a part of the vastness surrounding me.**

(Mi sono sentito parte della grandiosità che mi circondava).

1 2 3 4 5 6 7

***NEED TO SHARE (THE EXPERIENCE)***

**(Bisogno di Condivisione Dell’esperienza):**

1. **I feel the urge to share the experience with other people.**

(Sento il bisogno di condividere l’esperienza con altre persone).

1 2 3 4 5 6 7

1. **I have the feeling that, even though other people had been present, they would have lived the same I lived.**

(Sento che se anche altre persone fossero state presenti, avrebbero vissuto la stessa esperienza).

1 2 3 4 5 6 7

1. **I feel I am able to share a clear opinion regarding the experience.**

(Mi sento in grado di condividere un giudizio chiaro su quest’esperienza con altri).

1 2 3 4 5 6 7

1. **I do not desire to share this experience with other people. (R)**

(Non ho desiderio di condividere quest’esperienza con altre persone).

1 2 3 4 5 6 7

1. **I think I will not tell anyone what I saw. (R)**

(Penso che non racconterò quello che ho vissuto a nessun’altro).

1 2 3 4 5 6 7

1. **No one else could live what I lived. (R)**

(Nessun’altro potrebbe vivere quello che ho vissuto io).

1 2 3 4 5 6 7

***EXISTENTIAL SAFETY***

***(Senso di Sicurezza Esistenziale)***

1. **I felt safe despite the threaten I perceived.**

(Mi sono sentito sicuro nonostante la minaccia che percepivo).

1 2 3 4 5 6 7

1. **I felt overwhelmed by what I was observing. (R)**

(Mi sono sentito soverchiato da quello che stavo osservano).

1 2 3 4 5 6 7

1. **I felt safe in front of the danger.**

(Mi sono sentito protetto davanti al pericolo che vedevo).

1 2 3 4 5 6 7

1. **I felt at ease while I was watching the video.**

(Mi sentivo a mio agio durante la visione del video).

1 2 3 4 5 6 7

1. **I did not perceive the threaten as such.**

(Non percepivo che la minaccia fosse davvero reale).

1 2 3 4 5 6 7

**S1. Video Stimuli**

Please, click on this link to access video-stimuli used in this work: <https://www.dropbox.com/s/sls3sezt48ae4d9/Sublime%20Video%20Stimuli.zip?dl=0>

1. Item reverse [↑](#footnote-ref-1)
